# Understanding CAR T cell therapy and its role in ovarian cancer and peritoneal carcinomatosis from ovarian cancer

**DOI:** 10.3389/fonc.2023.1104547

**Published:** 2023-05-18

**Authors:** Víctor Domínguez-Prieto, Siyuan Qian, Pedro Villarejo-Campos, Cecilia Meliga, Sara González-Soares, Ismael Guijo Castellano, Santos Jiménez-Galanes, Mariano García-Arranz, Héctor Guadalajara, Damián García-Olmo

**Affiliations:** ^1^ Department of Surgery, Fundación Jimenez Diaz University Hospital, Madrid, Spain; ^2^ Department of Surgery, University Hospital Infanta Elena, Madrid, Spain; ^3^ Department of Surgery, Universidad Autónoma de Madrid, Madrid, Spain; ^4^ New Therapies Laboratory, Health Research Institute-Fundación Jiménez Díaz University Hospital (IIS-FJD), Madrid, Spain

**Keywords:** CAR-T cells, immunotherapy, solid tumor, ovarian cancer, peritoneal carcinomatosis, cell therapy, intraperitoneal chemotherapy, cytoreductive surgery

## Abstract

Ovarian cancer is the seventh most common cancer worldwide in women and the most lethal gynecologic malignancy due to the lack of accurate screening tools for early detection and late symptom onset. The absence of early-onset symptoms often delays diagnosis until the disease has progressed to advanced stages, frequently when there is peritoneal involvement. Although ovarian cancer is a heterogeneous malignancy with different histopathologic types, treatment for advanced tumors is usually based on chemotherapy and cytoreduction surgery. CAR T cells have shown promise for the treatment of hematological malignancies, though their role in treating solid tumors remains unclear. Outcomes are less favorable owing to the low capacity of CAR T cells to migrate to the tumor site, the influence of the protective tumor microenvironment, and the heterogeneity of surface antigens on tumor cells. Despite these results, CAR T cells have been proposed as a treatment approach for peritoneal carcinomatosis from colorectal and gastric origin. Local intraperitoneal administration of CAR T cells has been found to be superior to systemic administration, as this route is associated with increased tumor reduction, a more durable effect, protection against local relapse and distant metastases, and fewer systemic adverse effects. In this article we review the application of CAR T cells for the treatment of ovarian cancer and peritoneal carcinomatosis from ovarian cancer.

## 1 Introduction

Ovarian cancer (OC) is currently the seventh most common cancer worldwide in women, with 240,000 new cases diagnosed each year, and the second most common malignancy in women over age 40 years after breast cancer. It is the fifth leading cause of cancer-related death among women and the most lethal gynecologic malignancy due to the lack of accurate screening tools for early detection and the absence of clear symptoms until late-stage disease, when there is peritoneal involvement. Prognosis of OC is directly related to disease stage at diagnosis, with 5-year survival rates of 90% for stage I disease and dropping to 25% for metastatic disease (1). Even with optimal treatment, recurrence occurs in up to 80% of patients (2–4).

Treatment of OC is traditionally based on a combination of surgery and systemic chemotherapy. In patients with advanced stage OC, many of whom present peritoneal involvement, cytoreduction of all affected tissues is recommended where possible, including subsequent interval debulking surgery. Unfortunately, chemoresistance is one of the main causes of poor prognosis in patients with OC, as up to 80% of patients who initially respond to first-line therapy develop recurrent disease within 2 years despite multimodal treatment (5). Treatment outcomes remain poor for advanced ovarian malignancies, and there is a lack of effective therapeutic options in cases of relapse, which creates an urgent need for novel and effective therapeutic approaches.

CAR (chimeric antigen receptor) T cells are patients’ own T-lymphocytes obtained from whole blood and engineered using viral vectors to express a chimeric receptor against a selected surface antigen (TAA, tumor-associated antigen) without major histocompatibility complex (MHC) restriction. When the CAR recognizes a tumor-specific antigen, CAR T cells are activated, inducing tumor cell death (6).

CD19-targeted CAR T cells are a promising approach for the treatment of hematological non-solid malignancies, as they have achieved impressive clinical responses in almost 90% of patients. As a result, this therapy has been approved by the Food and Drug Administration (FDA) for treatment of acute lymphoblastic leukemia and diffuse large B cell lymphoma (7–10). Its role in the treatment of solid tumors remains unclear, however: disappointing results have been published as a result of the low capacity of CAR T cells to migrate to the tumor site, the protective tumor microenvironment, and the heterogeneity of surface antigens on tumor cells. However, combined approaches consisting of systemic chemotherapy and CAR T cells have a synergistic effect, since chemotherapy reduces tumor burden and plays an immunomodulatory role, thereby prolonging CAR T cell survival (11–13).

Recently, several authors have proposed using CAR T cells to treat peritoneal carcinomatosis (PC) of colorectal and gastric origin. In these conditions, local treatment with CAR T cells administered *via* peritoneal infusion has shown superior results to systemic administration, resulting in greater tumor reduction, a more durable effect, protection against local relapse and distant metastases, and fewer systemic adverse effects (14–17).

## 2 Overview of CAR T cells

### 2.1 Definition

The development of CAR T cells dates back to the late 1980s (6, 18). In general terms, these cells are lymphocytes that have been genetically modified to express chimeric receptors that enable the lymphocytes to recognize different surface antigens independently of MHC.

**Figure 1:**
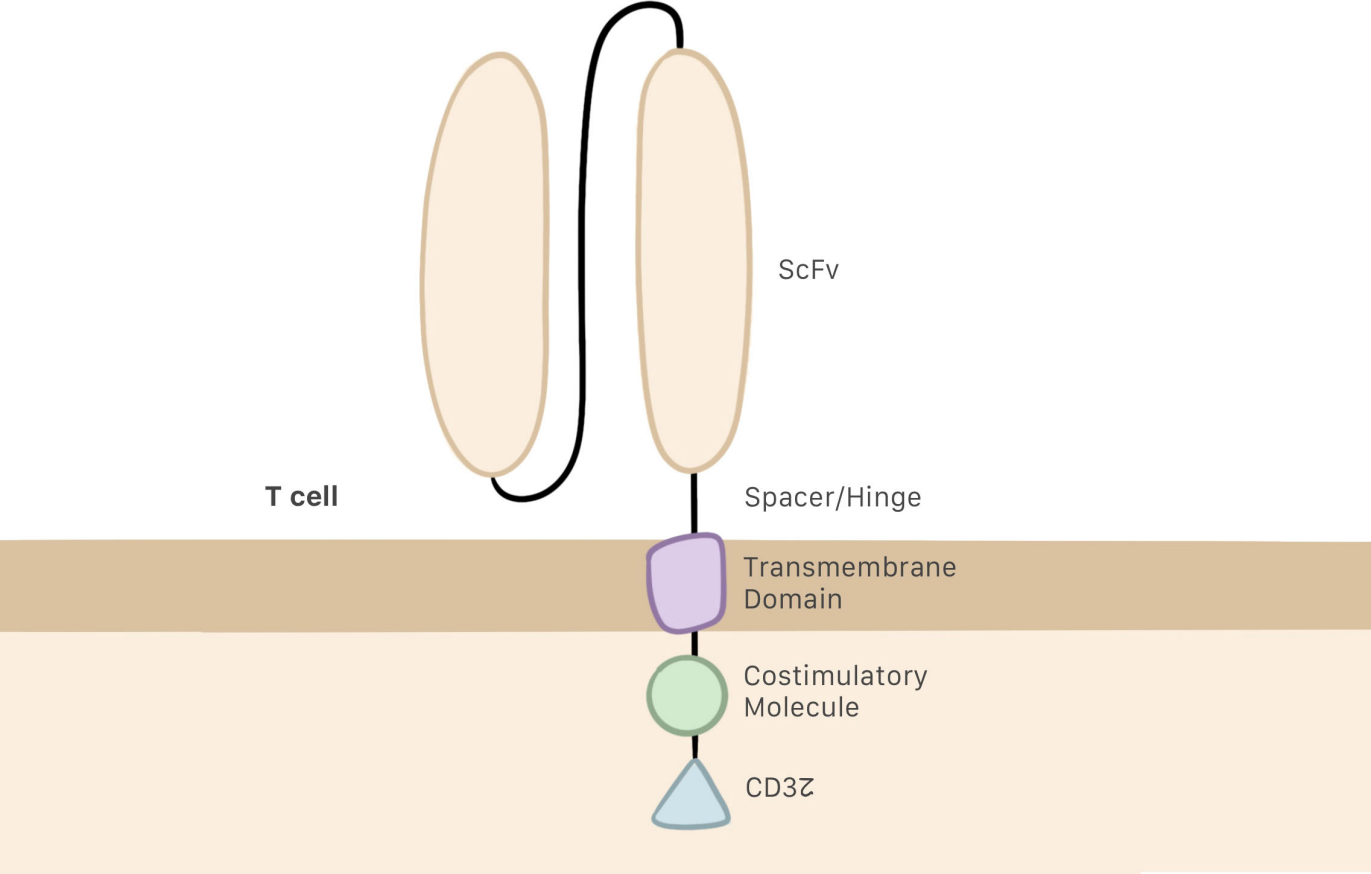
CAR T cell structure, including the ScFv, the hinge or spacer, and a transmembrane domain linking the intracellular regions, which are the CD3z domain and costimulatory molecules.

Several generations of CAR T cells have been developed over the years as changes have been progressively made to their structure. The earliest generations were formed by a single-chain variable fragment (ScFv), which enables the cell to recognize and bind to the target antigen; a hinge or spacer domain, which improves ScFv flexibility; and the CD3z intracellular domain, which activates the T cell. The problem with this generation was their inability to proliferate (6, 19, 20). To remedy this situation, second and third generations of CAR T cells included one or two costimulatory molecules in their intracellular domain, respectively (**Figure 1**) (21, 22). CD28 and 4–1BB are the most commonly employed costimulatory domains and make it possible to improve both the efficacy and persistence of CAR T cells (23, 24).

**Figure 2:**
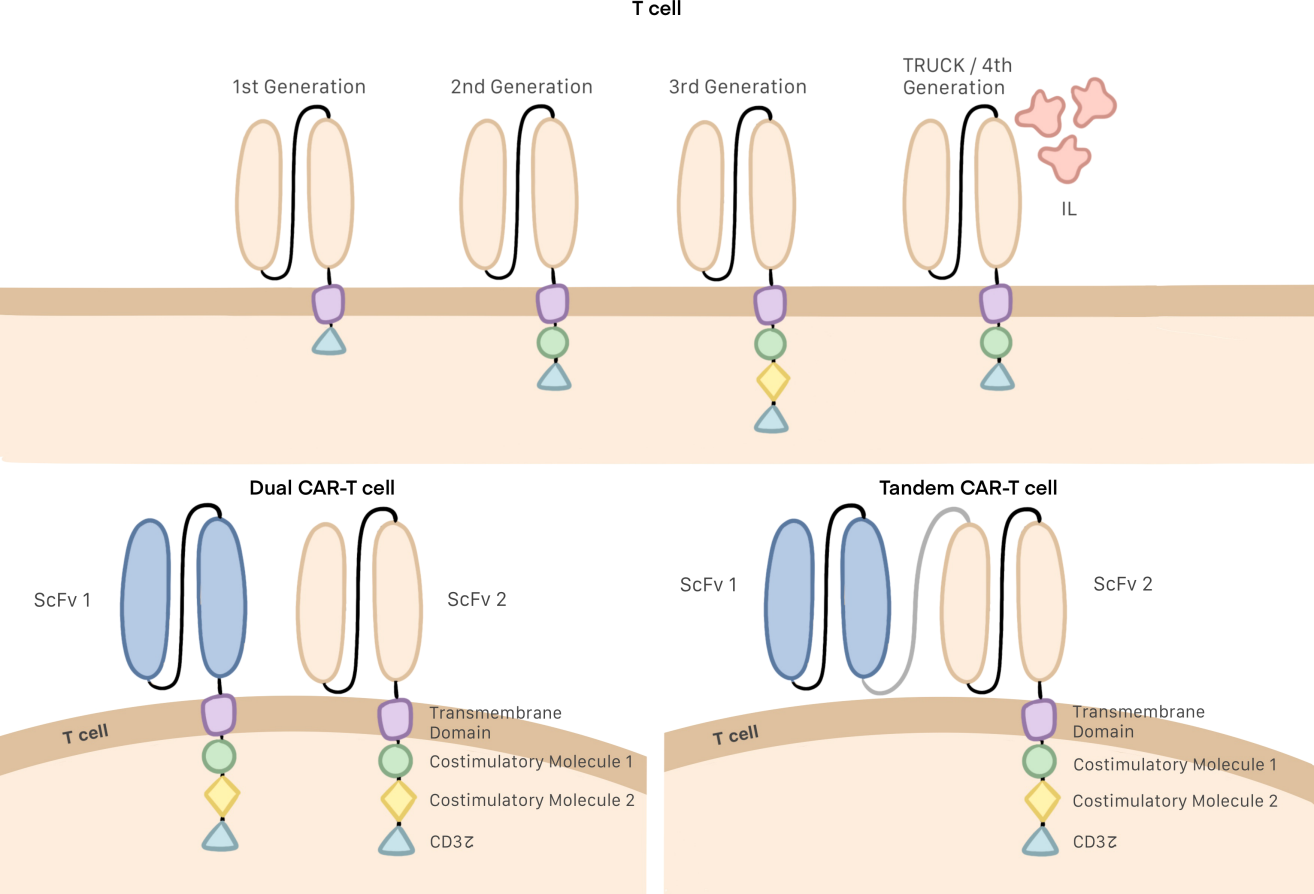
Generations of CAR T cells, Dual and tandem CAR T cells.

Despite these improvements in structure, it was discovered that the function of these third generation CAR T cells could be blocked by the immunosuppressive microenvironment. To prevent this, investigations are ongoing to design of a fourth generation of CAR T cells, also referred to as TRUCKs, or “T cells redirected for universal cytokine killing”. This generation of cells does introduces no specific structural modifications; rather, the improved function of TRUCKs stems from the production of proinflammatory cytokines deposited in the targeted tumor lesion, which can activate an innate immune cell response as well as block and escape the inhibitory effect of the tumor microenvironment (**Figure 2**) (22, 25).

Single-target CAR T cell therapy often leads to modest oncological outcomes and tumor recurrence due to the antigen heterogeneity and target antigen loss of tumor cells. Since mutational loss of the targeted epitope (downregulation or tumor escape) by tumor cells is a common immune-evasion strategy following single-target therapy, multiple antigen-targeting CAR T cells (dual CAR T cells and tandem CAR T cells) have been developed for improved specificity and effectiveness. It has been suggested that targeting multiple antigens simultaneously may improve the antitumor activity of CAR T cells by increasing antigen coverage and offsetting antigen loss (5, 26). Dual CAR T cells are T cells that bear two distinct CARs targeting different TAAs and can be activated when either of the two receptors recognizes the target. In contrast, tandem CAR T cells consist of a single intracellular signaling domain bound with two TAA-specific ScFvs and also can be activated with any of the two ScFvs (**Figure 2**) (27, 28).

### 2.2 CAR T cell production

Production of CAR T cells begins with a blood sample obtained from the patient. This sample must be centrifuged for extraction of white blood cells, and anticoagulants are added during the process to prevent blood clot formation. Then, the T cells are activated in a way that differs from physiological conditions, as during this process there is no interaction between the T cell receptor and the MHC. This activation is made possible by anti-CD3/CD28 coated magnetic beads as artificial antigen-presenting particles. Alternatively, T cells may be activated by using monoclonal antibodies or other artificial antigen-presenting cells (29).

**Figure 3:**
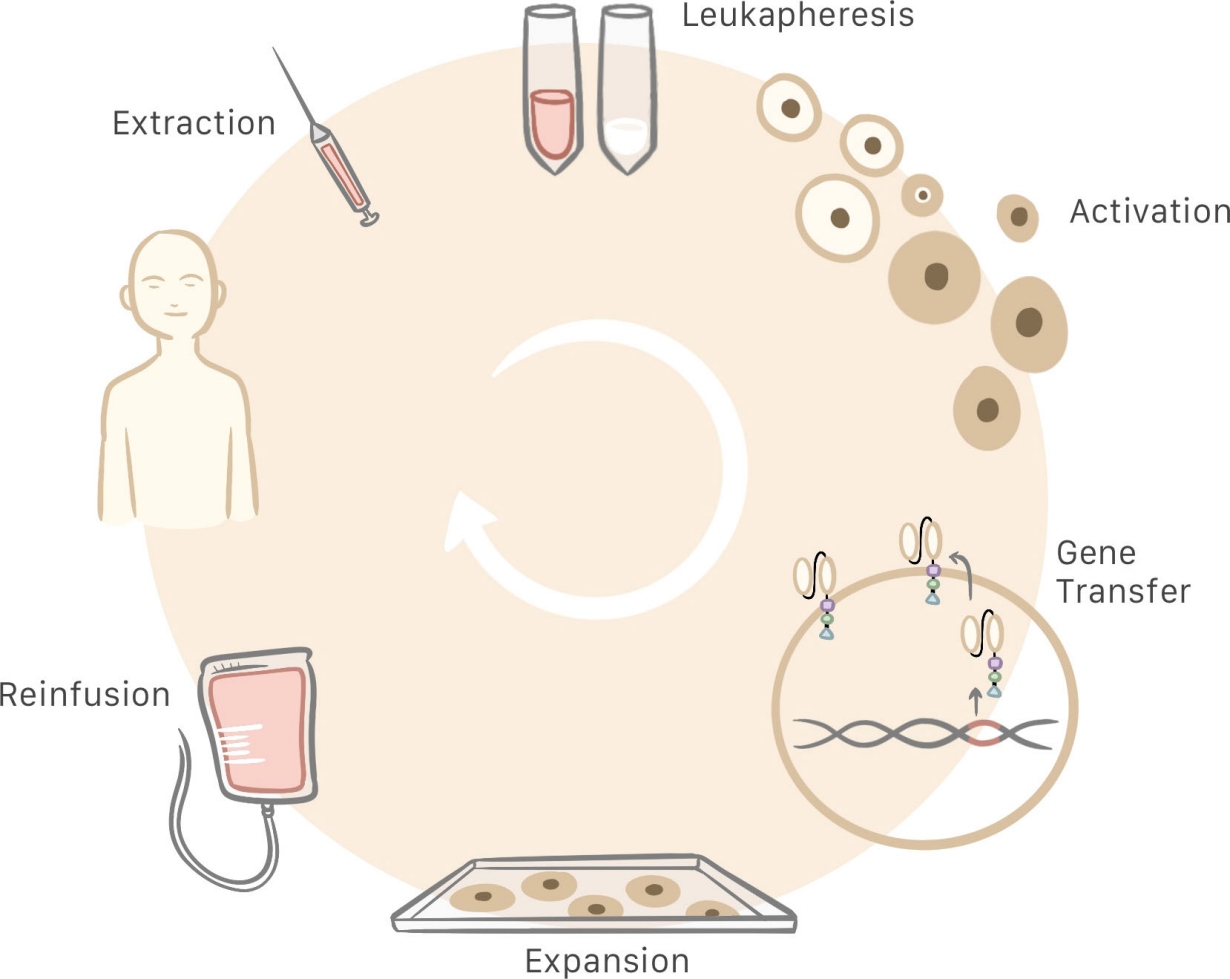
Patient blood is extracted and centrifuged to obtain the leukocytes. Then, T cells are selected and activated for subsequent insertion of the genes that generate the chimeric antigen receptor (CAR), which turns T cells into CAR T cells. Following cell expansion and quality control, the CAR T cells are infused into the patient.

Once activated, the T cell genome must be modified by inserting the gene that contains information needed to produce the CAR. Retroviral, and more specifically, lentiviral transduction is currently the most common method for transferring genes to CAR T cells. On conclusion of the activation and gene-transfer processes, cells are included in a medium supplemented with interleukin for expansion to a larger number of cells and subsequent quality control. If no contraindications are detected, the CAR T cells obtained can be infused into the patient (**Figure 3**) (13, 29, 30). Current manufacturing process is approximately 3 weeks (31). The total cost of the process is estimated at 300 000 USD and the price for commercialized CAR T cells are from 475.000 USD to 1.000.000 USD (29, 32, 33).

### 2.3 Adverse effects of CAR T cells

As in any other therapy or treatment, the use of CAR T cells has been related to adverse reactions, including some fatal outcomes. The severity of these effects and the toxicity of the treatment itself may be related to the type of CAR T cell, the target selected, the dose administered, or the use of conditioning before cell infusion (13, 34). Most of these side effects can be managed with conventional drugs. Also employing mRNA-transfected CAR T cells generates self-limited expression of CAR that reduces the duration of CAR T effect and therefore, decreasing potential toxicity.

#### 2.3.1 Cytokine release syndrome

Cytokine release syndrome (CRS) is the most common adverse effect in patients who undergo CAR T cell therapy, with an incidence of between 50 and 90% and a high mortality rate in some cases. CRS is the result of an over-production of cytokines caused by an excessive immune response as consequence of activated CAR T cells which stimulates other cells of the immune system (35, 36). This effect is related to treatment response and its absence raises doubts of the efficacy of the treatment (17, 35). CRS usually develops within a week of cell infusion and is caused by activation of T cells on a large scale, which produces several proinflammatory cytokines and may elicit a systemic inflammatory response. The most common symptoms of CRS are general malaise, nausea, and fever, and in some cases the condition progresses to acute respiratory distress, renal failure, cardiomyopathy and arrhythmias, coagulation disorder and, in the end, multiple organ failure (35). Treatment options vary from simple acetaminophen and fluid therapy to corticosteroids or even inotropic drugs in patients presenting hemodynamic instability (37). Several authors have suggested that CAR T cell-induced CRS is mediated by IL-6, IL-1, and nitric oxide produced by recipient macrophages, and can be attenuated by nitric oxide inhibitors, IL-6 antagonists such as tocilizumab, and IL-1 blockade by Anakinra (37, 38).

#### 2.3.2 Neurotoxicity

This adverse effect, known as CAR T cell-related encephalopathy or immune effector cell-associated neurotoxicity syndrome, can appear in 30 to 90% of cases. Neurotoxicity has only been described in CAR T cells engineered to target CD19 and CD20, and there is no evidence of neurotoxicity in solid tumors (13). Its cause its unknown, however it has been attributed to direct neurological damage or to an *off-tumor response* caused by the expression of CD19 in mural cells of the brain (13, 21, 39). It may also be related to high levels of cytokine released generating a systemic inflammation, affecting different tissues and promoting blood-brain barrier dysfunction allowing the arrival of immune cells including CAR T cells. Onset of this encephalopathy occurs around 5 or 7 days after cell infusion (34). Several symptoms have been described in this condition, such as headache, confusion, disorientation, focal neurological deficits and seizures, sometimes related to cerebral edema. In almost all of the cases described, symptoms are self-limited and resolve with no apparent long-term sequelae (35, 39, 40).

#### 2.3.3 “On-target, off-tumor” response

This adverse effect refers to an unwanted reaction of the immune system when a CAR T cell is activated by healthy tissues that express the target antigen, which may result in the destruction of healthy cells. The condition is more frequent when CAR T cell therapy is directed against solid tumors. To avoid this, it is essential to select a highly specific tumor antigen to target, though the CAR T cells must also have high specificity and affinity for the selected antigen (41, 42). An additional solution may be reached by establishing an appropriate dose of CAR T cells to administer or by choosing a more specific route of administration, such as intratumorally or intraperitoneally.

#### 2.3.4 Anaphylaxis

Anaphylaxis has been described following repeated CAR T cell therapy. This adverse effect is likely due to the development of human anti-mouse antibodies as a result of an initial intravenous administration of CAR T cells containing the murine ScFv domain. To mitigate the risk of developing anti-mouse ScFv antibodies, avoid this adverse effect and allow repeated administrations, the development of a fully human ScFv CAR binder domain has been proposed (43).

### 2.4 Barriers for CAR T cells in solid malignancies

Considering the promising results obtained with CAR T cells for the treatment of hematological malignancies, research is now focused applying them to solid tumors. Tumor cells have a unique cell surface protein and glycoprotein signature that distinguishes them from normal cells, thereby making them an attractive target for immunotherapy, including CAR T cell therapy (15). Multiple targets are currently being investigated for CAR T cell therapy in solid tumors, mainly overexpressed surface proteins and glycoproteins such as human epidermal growth factor receptor 2 (HER-2) for breast, lung, ovarian, and pancreatic cancer; carcinoembryonic antigen (CEA) for lung and digestive malignancies; MUC for pancreatic, gastric, and OC; epidermal growth factor receptor for glioblastoma and pancreatic cancer; and prostate-specific membrane antigen (PSMA) for prostate cancer, among others. However, CAR T cell therapy has shown less promising results for treating these other tumors than for hematological malignancies (13, 42, 44–47).

Unfortunately, solid tumors are biologically and molecularly different from non-solid malignancies in a number of aspects, and these differences may explain these unfortunate therapeutic results. TAAs in solid tumors are more heterogeneous than in hematological malignancies, which complicates efforts to find an optimal target. Achieving cell migration to the tumor site and penetration into the tumor is another difference that makes solid tumors less amenable to treatment with CAR T cells, as cells of solid tumors are protected by extracellular stroma and a hostile immunologic and metabolic microenvironment. Given these facts, numerous studies are under way to improve CAR T cell penetration into tissues and tumor cells as well as their persistence, antigen recognition, trafficking and, ultimately, their efficacy (48, 49).

#### 2.4.1 Heterogeneity of surface antigens (tumor-associated antigens) 

The lack of truly specific tumor antigens is one of the main obstacles to improving the effectiveness of CAR T cells against solid tumors (50). The antigens selected may be expressed not only by different tumors, but also by different tissues, including healthy ones, which may cause an *on-target, off-tumor response* as explained previously. In addition, antigen expression levels can vary across tumor locations or even in different parts of a single tumor due to the diversity of malignant cells, which makes it difficult to identify and select specific TAAs. Treatment of hematological malignancies derived from B cells have the advantage that all B-cell line expresses CD19 which makes it a perfect target. Anti-CD19-CAR T cells will destroy not only all tumor cells but also healthy B cells resulting in a B cell aplasia which makes the host more prone to opportunistic infections that nonetheless can be managed with immunoglobulin treatment (10). To avoid this response, a highly specific receptor for the tumor target must be selected, CAR T cells must have high affinity and specificity for the receptor chosen, and the CAR T cells must be administered at an appropriate dose (22, 41).

In order to circumvent antigen-negative tumor relapse (the presence of antigen-negative tumor cells that were not destroyed due to the lack of the target), some studies have described different methods to target multiple and different TAAs. One example is the research by Yan et al. (51), who suggested that double-target CAR T cells can avoid off-target effects. According to their approach, two different CARs are modified on T cells to recognize different antigens: one transmits the killing signal and the other provides the appropriate costimulatory signals. Both must bind to their corresponding antigen for the CAR T cell to be fully activated. This antigen-combination strategy could be useful to circumvent immune escape and could also augment the main CAR T cell antitumor effect (5, 26, 51). Thus, the development of Dual CAR T cells and Tandem CAR T cells may avoid tumor relapse.

#### 2.4.2 Downregulation, escape phenomenon, or antigen loss

As explained above, tumor antigen heterogeneity is one of the major hurdles facing CAR T cell therapy and can cause a phenomenon known as “escape” or antigen loss. Cells that present the selected TAAs are destroyed, though this also means that antigen-negative cells will not respond to CAR T cell therapy. When these cells survive and proliferate, the tumor develops resistance to CAR T cells as the number of antigens selected decreases. This phenomenon can also result from splicing events that modify the selected target, making it unrecognizable to the CAR T cell. Furthermore, changes to the survival and apoptosis mechanisms of the tumor cells, mainly in those cells presenting the antigen selected, can result in therapy resistance and lack of response to the treatment (15, 52, 53).

#### 2.4.3 CAR T cell migration, durability, and tumor trafficking

CAR T cell therapy is more limited against solid tumors, as CAR T cells are less capable of migrating to these tumor cells and penetrating the tumor tissue than when used to treat hematological tumors. In hematological tumors, both tumor and treatment cells (T cells) originate from hematopoietic tissue and tend to migrate to similar organs, which makes it easier for CAR T cells to migrate to the same area as the tumor cell. Furthermore, solid tumor tissues produce molecules such as cytokines or chemokines, which can interfere with or inhibit T cell migration to the tissue and prevent proper binding with the chemokine receptors presented on T cells (49, 50, 54). The need to penetrate the vascular endothelium in order to reach tumor tissue is another challenge limiting CAR T cell therapy against solid tumors due to alterations in the secretion of vascular-related factors (50).

To overcome these difficulties, local administration of CAR T cell therapy has been investigated, showing better results in cases of peritoneal dissemination than when the malignancy is restricted to a single organ. Also, CAR T cells can be genetically modified to express chemokine-specific receptors, improving migration and trafficking to the tumor cells (50, 54).

#### 2.4.4 Local microenvironment

The microenvironment surrounding the tumor is a formidable obstacle in CAR T cell therapy against solid tumors, as it acts as a triple barrier: physical, metabolic, and immunologic (13).

Different immunosuppressive cells and inhibitory factors are part of the tumor microenvironment, such as regulatory T cells (Tregs), myeloid-derived suppressor cells, tumor-associated macrophages, immunosuppressive cytokines (i.e., IL-10, TGF-B), or different ligands for tumor-expressed T cell inhibitory receptors (55, 56). These cells and factors contribute to tumor growth, angiogenesis, and metastasis and can block the effect of CAR T cell therapy (50, 51).

Tumor cells consume high levels of glucose, their main energy source. In the nutrition process, lactic acid is produced and can increase oxidative stress and inhibit T cell proliferation (48, 49, 57).

Tumor stroma is a physical barrier in the microenvironment that protects neoplastic cells from chemotherapy and the effects of CAR T cells. This extracellular matrix is formed by fibroblasts, myeloid cells, and high levels of collagen fibers, and may block CAR T cell penetration to the tumor (58, 59). One way to facilitate the effects of CAR T cells could be to destroy this matrix and its collagen fibers by administering collagenase locally prior to CAR T cell therapy for enhanced penetration into the tumor tissue (13, 60). In cases of peritoneal metastasis, which present high levels of collagen in their matrix, the ability of intraperitoneal collagenase to destroy the tumor stroma has been recently demonstrated in an experimental study (60). CAR T cell therapy could be more effective if administered after local collagenase to overcome this physical barrier.

## 3 Generalities of ovarian cancer

### 3.1 Current status in ovarian cancer

Ovarian carcinomas can be considered relatively immunogenic tumors. The high quantity of endogenous tumor-infiltrating T cells observed within the tumor microenvironment is related to enhanced survival after treatment (55). Nevertheless, high levels of immunosuppressive regulatory T cells within the tumor microenvironment, which abrogate the antitumor activity of effector T cells, are related with a worse prognosis. These findings suggest an endogenous effector T cell response against tumor cells and a possible therapeutic benefit of CAR T cell therapy targeted against tumor cell antigens (61).

The peritoneum is a common site of metastasis in OC due to the intraperitoneal location of the ovaries. Multimodal treatment for PC from OC is based on systemic chemotherapy and cytoreduction surgery of all affected tissues with subsequent interval debulking surgeries. Surgery is considered optimal if residual tumor nodules have a maximum diameter or thickness of less than 1 cm; otherwise, surgical treatment is associated with worse oncologic outcomes. Intravenous taxane/carboplatin and liposomal doxorubicin/carboplatin-based neoadjuvant chemotherapy are indicated to reduce tumor burden before surgery and as adjuvant chemotherapy after surgery. Novel therapies such as poly ADP-ribose polymerase inhibitors, which include olaparib and rucaparib, also have demonstrated efficacy in treating OC when used in conjunction with chemotherapy (1).

Although 70% of patients with OC show a complete response to treatment following debulking surgery and adjuvant chemotherapy, nearly 70% of these patients develop recurrent disease, most in the form of PC. Patients with first disease recurrence experience subsequent recurrences, resulting in shorter recurrence-free survival and median interval of recurrence. Several studies suggest better survival rates with administration of intraperitoneal chemotherapy in addition to cytoreductive surgery compared to systemic chemotherapy plus cytoreductive surgery, both for advanced and recurrent disease (62–65).

Hyperthermic intraperitoneal chemotherapy (HIPEC) combines the effect of intraperitoneal chemotherapy with the three main effects of heat: the cytotoxic effect of lysosome activation, the synergistic effect with some anti-mitotic agents, and the penetration effect, which increases the sensitivity of tumor cells to chemotherapy. Even if results of these studies are promising, findings on the efficacy and morbidity associated with HIPEC have raised doubts as to this approach as a standard of care for both primary and recurrent OC (66). To date, there is still no consensus on the ideal temperature of hyperthermia, the most suitable drug for intraperitoneal chemotherapy, or the optimal protocol for agent delivery in recurrent OC.

For colorectal and gastric PC, peritoneal delivery of CAR T cells has been found to be superior to systemic administration as evidenced by better CAR T cell trafficking and infiltration, greater tumor reduction, a more durable effect, enhanced protection against local relapse and distant metastases, and fewer adverse systemic effects (14, 16). This protection conferred by local peritoneal administration against distant metastases is not explained by the direct action of CAR T cells, but rather by a phenomenon resembling the abscopal effect. This effect, which usually occurs in radiotherapy, results from tumor antigen release by the tumor cells destroyed by CAR T cells, which enables antigen cross-presentation by dendritic cells and elicits an immune response against different tumor antigens not targeted by CAR T cells (14, 67, 68). Moreover, the release of cytokines stimulates the innate immune response (17).

### 3.2 Histopathologic types

OC is a histologically heterogeneous malignancy comprising three histopathologic types (epithelial, germ cell, and sex cord-stromal) and several subtypes, creating a heterogeneous landscape of possible TAAs. Both germ cell and sex cord-stromal OCs are rare, making up 3% and 2% of all malignancies of the ovaries, respectively. Epithelial ovarian cancer (EOC) accounts for approximately 90% of OC cases and is divided into two main categories. Type I tumors are usually less lethal and typically present as low-stage disease. Pathogenically, they are thought to be caused by continued ovulation cycles, persistent inflammation, and endometriosis. In contrast, type II tumors are commonly diagnosed in advanced stages and are thus associated with more fatal outcomes and often linked to genetic mutations. EOC has four primary histologic subtypes, from the most to the least common: serous, endometrioid, clear cell, and mucinous. Both endometrioid and clear cell carcinomas are often diagnosed in early stages, resulting in a relatively good prognosis; mucinous histology can be associated with metastasis of gastrointestinal origin. The serous subtype is further divided into two categories: high-grade serous carcinomas, making up 90% of all serous tumor types, with more fatal prognosis and a 10-year mortality rate of 70%; and low-grade serous carcinomas, which have a better prognosis and usually a younger age at diagnosis (1, 69).

### 3.3 CAR T cell targets in ovarian cancer

The major bottleneck for CAR T cell therapy in solid tumors is the limited availability of specific TAAs, whose homogeneous overexpression on malignant cells and minimal to no expression on healthy cells can minimize “off-tumor, on-target” toxicity. Ovarian tumor cells have a unique aberrantly glycosylated cell surface protein signature that distinguishes them from normal epithelia and therefore represent an attractive target for immunotherapy, including CAR T cell therapy (23). The wide range of possible TAAs is a rich source of attractive CAR T cell targets due to their overexpression in OC cells; we describe some possible TAAs below.

#### 3.3.1 Mucin 16

Mucin 16 (MUC16), the glycoprotein with the highest molecular weight of the mucin family, plays a critical role in maintaining intracellular homeostasis and protecting the epithelium. Its structure consists of a single membrane-spanning domain, a cytoplasmic tail, an extensive extracellular N-terminal domain, and a tandem repeat sequence. The extracellular region has a cleavage site distal to which there is an external domain that includes 16-20 tandem repeats of 156 amino acids, each with potential glycosylation sites. When cleavage occurs, the MUC16 ectodomain (MUC16ecto) stays on the cell surface while the N-terminal domain, called CA-125 (a well-known circulating marker of early-stage disease, also routinely used for monitoring the clinical course of OC patients), is released into the peripheral bloodstream. Studies have shown that over 80% of EOCs overexpress MUC 16 (MUC16ecto and CA125), with female patients having elevated levels of serum CA-125 (>65U/ml) and MUC16ecto expressed on their tumor cells. MUC16ecto is also expressed on normal tissues including those of the uterus, endometrium, fallopian tubes, ovaries, and, at lower levels, on the serosa of the abdominal and thoracic cavities, making it an attractive TAA for CAR T cell therapy (55). After cleavage, however, part of the extracellular domain of MUC16 is secreted as CA-125 fraction. To date, all reported monoclonal antibodies directed toward MUC16 bind to epitopes present on the CA-125 fraction of the glycoprotein, and none is known to bind to the retained extracellular MUC16ecto fraction of the antigen; this retained fraction with independent pro-oncogenic properties remains on the tumor cell surface and therefore represents the most attractive target for immune-based therapies in patients with advanced EOC (61, 70, 71).

#### 3.3.2 Mesothelin

Mesothelin (MSLN) is a glycoprotein anchored to the plasma membrane by a glycophosphatidyl inositol domain; after cleavage of the protein, a C-terminal fragment remains attached to the membrane while a soluble N-terminal fragment called megakaryotic potentiating factor is released. MSLN is expressed at low levels in normal mesothelial tissues, such as those of the pleura, peritoneum, and pericardium, and minimally on the epithelial surface of the trachea, ovaries, fallopian tubes, and tonsils; apparently, this molecule has a nonessential biological function, since MSLN knockout mice exhibit normal features. In contrast, aberrant MSLN expression plays a key role in cancer proliferation, invasion, and metastasis formation through the activation of the PI3K, ERK, and MAPK signaling pathways and resistance to apoptosis. MSLN is overexpressed in 90% of patients with epithelioid malignant pleural mesothelioma, 69% of lung adenocarcinomas, 60% of breast cancer cases, 46% of esophageal cancers, but also on pancreatic ductal adenocarcinoma and OCs (72). Its wide overexpression in multiple solid cancers makes MSLN a promising target for immune-based therapies such as CAR T cell therapy, having shown favorable safety profiles and clinical activity in early clinical trials (23, 43, 72).

#### 3.3.3 Folate receptor 1

The alpha isoform folate receptor alpha (FRα), also known as gene folate receptor 1 (FOLR1), is another glycophosphatidylinositol-anchored membrane protein. It has low expression in normal tissues and is overexpressed in 90% of OC, where it has been shown to significantly correlate with histological grade and stage. FRα also facilitates resistance to chemotherapy in OC patients, since its higher expression on tissues is associated with a lower response rate to mitotic agents; furthermore, protein expression on the tumor surface is not affected by chemotherapy, which makes it an ideal TAA for targeted immunotherapy treatment. Studies have shown that by targeting FOLR1 or MSLN alone there is a 48 to 76% likelihood of near-complete tumor elimination, and simultaneous targeting of these two proteins brings the killing of tumor cells up to 88% (5, 71, 73).

#### 3.3.4 Tumor-associated glycoprotein 72

Tumor-associated glycoprotein 72 (TAG72), the truncated onco-fetal sialyl-Tn (STn) hapten located on multiple cell surface glycoproteins, is created due to an aberrant glycosylation pathway in cancer cells. TAG72 can be found overexpressed in several adenocarcinomas, including breast, colon, pancreatic, prostate cancers and in 90% of EOCs, while it is practically absent from normal tissues. Increased TAG72 expression has been directly linked to poorer prognosis, therefore making this glycoprotein an attractive option for CAR T cell therapy in advanced EOC. High levels of TAG72, MUC1, and MUC16 expression have been found in tissue samples from EOC patients, with nearly 100% exhibiting simultaneous presence of all three antigens on the affected tissue (15, 26).

#### 3.3.5 Programmed cell death-1

Programmed cell death-1 (PD1) and its ligand, programmed cell death-ligand inhibitor (PDL1), are immunosuppressive molecules of the CD28/cytotoxic T lymphocyte-associated antigen-4(CTLA-4) family, and the role of PD1 is to inhibit T cell immunity. Studies showed that cytokines secreted by T cells, like IL-10 and IFN-γ, induce the expression of specific CTLA ligands such as PD1 on OC cells; additionally, PD1 stimulates the expression of inhibitory receptors on the T cell surface and binds to them, regulating T cell repositioning and affecting the immune escape mechanism (70, 71).

#### 3.3.6 CD44

CD44, also referred to as P-glycoprotein 1, is a transmembrane glycoprotein initially identified as a receptor for hyaluronan (HA) and a ubiquitous component of the extracellular membrane implicated in the cell-cell and cell-matrix interactions, associated with metastasis promotion. CD44 is highly expressed in a variety of cancers, including OC. Binding between CD44 and HA mediates tumor cell adhesion to the peritoneum, hence promoting intraperitoneal OC spread. A significant correlation has been found between CD44 expression and disease-free survival and overall survival in OC patients. *In vitro* and *in vivo* studies showed that downregulation of CD44 expression reduces the migratory and invasive potential of OC cells, halting tumor growth as well as peritoneal dissemination; by contrast, overexpression of CD44 has been found in relapsed/recurrent tumors of xenograft mice models treated with taxane-based chemotherapy. Overall, CD44 is a promising target molecule for immunotherapy in OC (74).

#### 3.3.7 5T4 oncofetal antigen

The 5T4 oncofetal antigen is an N-glycosylated transmembrane protein found both on human on trophoblasts and cancer cells. The protein is highly expressed on the surface membrane of a variety of different cancers, including OC, and is rarely found in normal adult tissues. 5T4 plays a role in tumor development and spread, and its expression correlates with poorer clinical outcomes. The selective tumor expression pattern of 5T4 together with its tumor-initiating and tumor-spreading role makes this molecule an attractive target for CAR T cell therapy (71, 75).

#### 3.3.8 L1 cell adhesion molecule

The L1 cell adhesion molecule (L1-CAM) is a transmembrane glycoprotein that normally promotes the adhesion and motility of cells of the nervous system. It is overexpressed in various tumors, including OC, and correlates with poor prognosis and late-stage disease. L1-CAM expression promotes tumor invasion and progression through increased IL-1β production and NF-κB activation. Its absence in normal ovarian tissues qualifies this molecule as a target for immunotherapy (71, 76).

#### 3.3.9 Follicle stimulating hormone receptor

The follicle stimulating hormone receptor (FSHR) is selectively expressed in ovarian granulosa cells and different histological types of OC, including 50-70% of serous ovarian carcinomas, but not in other healthy tissues apart from ovaries. Since oophorectomy is a standard treatment for ovarian carcinomas, targeting FSHR should not cause on-target, off-tumor toxicity in healthy tissues in patients with OC (77).

## 4 CAR T cell therapy in ovarian cancer and peritoneal carcinomatosis of ovarian origin

CAR T cells have become a promising therapeutic approach for the treatment of hematological malignancies, and impressive clinical responses have been reported in acute lymphoblastic leukemia and diffuse large B cell lymphoma. Propelled by these initial findings, multiple preclinical studies using CAR T cells have aimed to clarify their role in OC with PC. However, this research has obtained less promising results compared to studies with hematological malignancies due to such limitations of CAR T cell therapy in solid tumors as the low capacity of CAR T cells to migrate to the tumor site, the protective tumor microenvironment and stroma, the heterogeneity of surface antigens, and the antigen loss displayed by tumor cells following single-target therapy, which leads to tumor recurrence. Research employing CAR T cells *in vitro*, *in vivo* and in clinical trials are summarized in **Table 1**.

**Table 1:** Antigen used in CAR T cell therapy for ovarian cancer *in vitro, in vivo* and in randomized clinical trials.

Target antigen	*In vitro*	*In vivo*	Ongoing RCT
**MUC16**	Yes (55, 61, 70)	Yes (55, 61, 70)	NCT05239143 NCT04025216
**MSLN**	Yes (5, 23, 43, 78, 79)	Yes (5, 23, 43, 78, 79)	NCT03916679 NCT03799913 NCT04562298 NCT03814447 NCT04503980 NCT05372692 NCT01583686 NCT03054298 NCT02580747 NCT05568680 NCT02159716
**FOLR1**	Yes (5, 80)	Yes (5, 80)	No
**TAG72**	Yes (15, 26)	Yes (15, 26)	NCT05225363
**PD1**	Yes (70)	Yes (70)	No
**CD44**	No	No	No
**5T4 oncofetal antigen**	Yes (75)	Yes (75)	No
**L1-CAM**	Yes (76)	Yes (76)	No
**FSHR**	Yes (77, 81, 82)	Yes (77, 81, 82)	NCT05316129

MUC16, mucin 16; MSLN, mesothelin; FOLR1, folate receptor 1; TAG72, tumor-associated glycoprotein 72; PD1, programmed cell death-1; CD44, cluster of differentiation 44; L1-CAM, L1 cell adhesion molecule; FSHR, follicle stimulating hormone receptor; RCT, randomized clinical trial.

### 4.1 Conventional CAR T cell therapy

For treatment of OC, Schoutrop et al. employed second-generation MSLN-directed CAR T cells (with CD28 and 4–1BB costimulatory domains) in orthotopic murine models of ovarian serous carcinoma. In the study, intravenous CAR T cell therapy significantly augmented mouse survival. Persistent remissions were induced in some cases, though the treatment effect was generally transient and resulted in delayed tumor regrowth, thereby demonstrating that coinhibitory pathways hamper CAR T-cell persistence in the ovarian tumor microenvironment. After CAR T cell therapy, MSLN expression in tumor cells decreased progressively. High levels of cytokines produced by CAR T cells such as IFN gamma, GrzA, GrzB, TNF, Fas, FasL, IL10, and TGFα were related to significatively prolonged survival (23). Similar results were achieved by another group, who employed third-generation MSLN-targeted CAR T cells (with CD28 and 4-1BB costimulatory domains) in an experimental mouse model. *In vitro*, MSLN-targeted CAR T cells specifically killed MSLN-positive ovarian tumor cells. In mice, intravenous infusion of CAR T cells resulted in a significant reduction of tumor size compared to control animals (78).

Hung et al. generated anti-human MSLN mRNA CAR for treating OC with PC and demonstrated that these CAR T cell can destroy ovarian tumor cells *in vitro* and in a murine model. Intraperitoneal CAR T cell administration showed dose-dependent inhibition of tumor growth and improved survival compared with control mice. However, since these CAR T cells are transiently transfected by mRNA, they require repeat dosing for improved efficacy of tumor elimination (43).

*Bordoloi et al.* (81) *and Urbanska et al.* (82) demonstrated high antigen-specific cytotoxicity of FSHR-targeted CAR T cells against FSHR+ OC cell lines *in vitro*. A significant reduction in subcutaneous tumor volume and suppressed tumor growth, respectively, following intraperitoneal or intravenous injection of CAR T cells were achieved *in vivo* in murine models of subcutaneous OC.

*Perales-Puchalt et al.* (77) tested the safety and effectiveness of FSHR-targeted second-generation CAR T cells against peritoneal or subcutaneous OC implants, both *in vitro* and *in vivo*. *In vivo*, anti-FSHR T cells killed FSHR+ cultured OC cells in a dose-dependent manner. In a xenograft orthotopic subcutaneous and peritoneal model of OC in immunocompetent mice, intraperitoneal administration of FSHR-targeted T cells demonstrated significant therapeutic response, even tumor rejection, and an increased survival without on-target, off-tumor toxicity. Persistence of CAR T cells as memory lymphocytes was observed. Notably, chimeric receptors enhanced pre-existing endogenous T cell-dependent antitumor immunity against OC cells.

Song et al. (80) developed a fully human CAR composed of the human C4 FRα specific ScFv for treatment of mice with PC from OC, aiming to avoid transgene immunogenicity and “on-target, off-tumor” toxicity of CARs containing mouse-derived ScFvs and to facilitate the persistence and activity of αFR-targeted CAR T cells in humans. These first- and second-generation αFR-targeted CAR T cells specifically recognized and efficiently lysed αFR-expressing ovarian tumor cells *in vitro.* Although the human C4 Fab fragment showed five-fold less binding affinity than the murine ScFv, intravenous infusion of anti-αFR CAR T cells significantly delayed tumor progression of established human αFR-positive tumors in xenogeneic mouse models of advanced subcutaneous and intraperitoneal OC. Second-generation CAR T cell therapy, which included a CD27 costimulatory domain, was superior to treatment with first-generation CAR T cells. The authors concluded that anti-αFR CAR T cells with fully human CAR showed lower antigen affinity but similar antitumor activity with a less immunogenic response, resulting in a lower risk of “on-target” toxicity because of the lower level of proinflammatory cytokine production when compared with anti-αFR CAR T cells carrying murine CAR.

Chekmasova et al. (61) employed first- and second-generation CAR T cells targeted to the extracellular domain of MUC16 (MUC-CD) in an *in vitro* and *in vivo* peritoneal ovarian carcinoma murine model. MUC-CD-targeted CAR T cells showed efficient MUC-CD-specific cytolytic activity against ovarian carcinoma cells *in vitro*. *In vivo*, intravenous and intraperitoneal administration of MUC-CD-targeted CAR T cells in an orthotopic human MUC-CD+ peritoneal ovarian carcinoma murine model also showed delayed progression or full eradication of the disease, with statistically significant increased survival when compared to untreated control mice. The authors observed no statistically significant difference in survival when comparing first- and second-generation CAR T cells. Both intravenous and intraperitoneal administration of second-generation CAR T cells evidenced significantly enhanced survival when compared with untreated animals as well as equivalent antitumor efficacy. Appropriate trafficking of intravenously infused second-generation CAR T cells to the peritoneum was confirmed, with a marked population of CAR T cells within peritoneal tumor deposits 1 day after administration. CAR T cell therapy initially eradicated most evident disease in all treated mice, but disease relapse was observed in 75% of the specimens. CAR T cells were still detectable in peritoneal washings 28 days after infusion, although with a decreased quantity.

Another group demonstrated the efficacy of second-generation CAR T cells with a humanized anti-human TAG-72 scFv antigen-binding domain and a 4-1BB intracellular costimulatory signaling domain (TAG72-BBζ) in both *in vitro* and *in vivo* murine experimental models of peritoneal ovarian tumors. *In vitro*, TAG72-BBζ CAR T cells demonstrated strong antigen-dependent cytotoxicity against multiple TAG72-expressing human OC cell lines and epithelial cells derived from patient cancer-related ascites. Local intraperitoneal administration of TAG72-BBζ CAR T cells demonstrated significant antitumor activity, eliminated antigen-positive disease, significantly reduced tumor growth, and increased overall survival in an *in vivo* xenograft murine model of PC from OC, while intravenous administration was ineffective in controlling disease. Tumor control was further improved with repeat intraperitoneal infusions of CAR T cells. Nevertheless, they observed that early recurring tumors displayed an antigen scape phenomenon, with decreased TAG72 expression related to a lack of CAR T cell persistence intraperitoneally. This can be explained by the transient internalization of TAG72 following exposure to CAR T cells and pre-existing tumor cells with lower TAG72 expression that are not recognized by CAR T cells (15).

A similar study employed second-generation CAR T cells (CE7R+ T cells) targeting the CE7-epitope of L1-CAM which is aberrantly expressed in ovarian malignancies but absent from normal ovarian tissue, in conjunction with a CD28 costimulatory domain. *In vitro* studies showed that CE7R+ T cells exhibited robust cytolytic activity against L1-CAM+ OC cell lines, producing significant amounts of proinflammatory cytokines, doing so without targeting L1-CAM negative normal ovarian cells. Intraperitoneal administration in a human-murine xenograft model of peritoneal ovarian tumors induced significant peritoneal tumor reduction, inhibited ascites development, and significantly lengthened survival in mice versus control animals. However, CE7R+ T cells did not completely eradicate tumor growth. Recurrent or residual tumors had markedly reduced L1-CAM/CE7 expression, suggesting antigen loss as a potential mechanism of tumor escape (76).

Oncofetal TAA 5T4-targeted CAR T cell therapy was tested by Guo et al. (75). They developed second-generation human CAR T cells that showed specific concentration-dependent cytotoxicity against 5T4+ cells and significantly higher secretion of cytotoxic cytokines *in vitro*. In a peritoneal xenograft mouse model, intraperitoneal administration of anti-5T4 CAR T cells showed significant and effective antitumor activity, as they eradicated peritoneal tumors in a short time without recurrence and prolonged survival. In a subcutaneous xenograft mouse model, intravenous anti-5T4 CAR T cell therapy also controlled tumor growth, though its effect was less pronounced than the peritoneal model. This difference can be explained by difficulties in cell trafficking and the lower quantity of effector CAR T cells reaching the tumor site following intravenous infusion compared to intraperitoneal administration.

Research has found intraperitoneal administration to be superior to systemic administration for treating PC from OC with such benefits in mice as improved CAR T cell trafficking and infiltration, greater tumor reduction, a more durable effect, and increased survival. Repeat intraperitoneal administration further improves these results.

The addition of costimulatory domains, such as 4-1BB and/or CD28, seems to improve cytotoxicity and CAR T cell persistence. Third-generation CARs, which include two costimulatory domains, appear to have more potent antitumor activity than first- and second-generation cells (with zero and one costimulatory domain, respectively) (75, 80). The 4-1BB domain has been suggested to be superior to CD28, as it shows longer persistence of T cells *in vivo*, CAR T cell activation, and durability (75).

### 4.2 Dual CAR T cell therapy

Multi-target CAR approaches may also enhance the persistence of CAR T cells within ovarian tumor tissue. Targeting multiple antigens may improve the antitumor activity while decreasing the probability of antigen loss or tumor escape.

In a non-PC model of OC, third-generation tandem CAR T cells targeting both FOLR1 and MSLN have also been studied. These dual-target CAR T cells showed higher cytotoxicity against ovarian tumor cells and secreted higher levels of cytokines than single-antigen CAR T cells *in vitro*. *In vivo*, intravenous dual CAR T cell therapy exhibited significantly stronger antitumor activity, markedly reduced tumor volume, and prolonged survival in a xenograft mouse model of subcutaneous OC when compared with single-target CAR T cells, reducing tumor antigen escape and increasing T cell functionality. Moreover, higher infiltration and persistence of T cells in tumor samples was observed for dual CAR T cells (5). Similarly, Shu et al. (26) developed second-generation dual CAR T cells targeting the ovarian tumor antigens TAG-72 and CD47. However, as CD47 is also expressed by normal cells, including T cells, the authors developed a truncated CD47 CAR without intracellular signaling domains to avoid normal tissue damage and T cell fratricide. This CD47-truncated CAR enhances the binding affinity of CAR T cells for TAG72+ cancer cells. The CD28 and 4-1BB costimulatory domains were compared *in vitro*: CD28-costimulated CAR T cells showed significantly greater cytotoxicity, with a quicker T cell response following antigen exposure and a similar cytokine release than 4-1BB costimulated cells. Nevertheless, 4-1BB-costimulated dual CAR T cells showed no cytotoxicity against CD47+ normal cells, so the authors employed these 4-1BB-costimulated cells in *in vivo* investigations. Dual CAR T cells co-expressing TAG-72 and CD47-truncated CARs showed stronger cytotoxicity *in vitro* when compared to single-target CAR T cells, especially against ovarian tumor cells expressing low levels of TAG-72. In a subcutaneous murine model of OC, intravenous infusion of dual CD47- and TAG-72-targeted 4-1BB-costimulated CAR T cells delayed tumor growth compared to single TAG-72-targeted CAR T cells.

Li et al. (70) investigated the therapeutic effect of second-generation dual MUC16 and PDL1-targeted CAR T cells in a murine xenograft model of peritoneal OC. Previous studies demonstrated that when PD1 co-expressed with anti-MSLN on CAR T cells, the PD1 chimera strengthened the effector activity of CAR T cells in mice. Dual CAR T cells showed similar ability and cytotoxicity against OC cells when compared to single CAR T cells *in vitro*, as well as comparable cytokine secretion capacity. Intraperitoneal administration of dual CAR T cells exhibited significant regression of OC cells with a more potent therapeutic effect than single-target CAR T cells and significantly prolonged survival in mice compared with single-target control groups. Dual CAR T cell therapy was demonstrated to be two to four times more effective than single CAR T cell therapy in terms of survival, avoiding on-target, off-tumor toxicity and CRS, improving target specificity, and avoiding immune escape. These different results between *in vitro* and *in vivo* assays are attributable to the local tumor microenvironment.

These results suggest that targeting multiple antigens simultaneously by means of dual CAR T cell therapy may improve the antitumor activity of CAR T cells by increasing antigen coverage and avoiding downregulation of targeted antigens.

### 4.3 Cytokine-secreting CAR T cells

Activation of CAR T cells is usually inhibited by the tumor microenvironment, as is commonly seen in the endogenous antitumor immune response. To avoid this limitation, CAR T cells can be modified to secrete stimulatory cytokines that promote the antitumor immune response. IL12 is a stimulatory cytokine that enhances CD8+ T cell function, increases secretion of IFN-γ, improves cytotoxic capacity, reactivates anergic tumor-infiltrating lymphocytes, inhibits Treg-mediated suppression of effector T cells, recruits NK cells to the tumor site, and inhibits IL-10 and TGF-β secretion, all of which suggests that IL12 could modulate the inhibitory microenvironment of solid tumors (55).

Koneru et al. (55) developed second-generation CAR T cells expressing a CAR against the extracellular domain of MUC16 and modified to secrete IL12. These IL12-secreting MUC16-targeted CAR T cells showed enhanced proliferation and significantly higher IL12 and IFN-γ secretion compared to anti-MUC16 non-IL12-secreting cells, but similar cytotoxicity against MUC16+ OC cells *in vitro*. Working with a peritoneal xenograft mouse model of OC, the authors demonstrated that intraperitoneal injection of IL12-secreting CAR T cells resulted in significantly enhanced antitumor efficacy with no evidence of disease after treatment in any case, increased survival of mice, prolonged persistence of T cells, and higher systemic IL12 and IFN-γ secretion when compared to non-IL12-secreting CAR T cells. Both intravenous and intraperitoneal administration of CAR T cells prolonged animal survival, though intraperitoneal administration was found to be significantly more effective.

Similar results were observed by Pang et al. for ovarian, hepatocellular, and pancreatic carcinomas. They observed that IL7- and CCL19-secreting glypican-3- and MSLN-targeting CAR T cells had an enhanced capacity for expansion and migration and augmented chemotaxis *in vitro* when compared to non-cytokine-secreting CAR T cells. In xenograft mouse models of advanced ovarian, hepatocellular, and pancreatic carcinomas with glypican-3 or MSLN expression, these cytokine-secreting CAR T cells showed enhanced antitumor activity, higher cell levels in peripheral blood, and increased infiltrating CAR T cells in tumor tissue compared to non-cytokine-secreting CAR T cells, and significantly suppressed tumor growth (79).

Fourth-generation cytokine-secreting CAR T cells have therefore been proposed to modulate the inhibitory effect of the tumor microenvironment, inhibit the suppression of effector T cells, and promote the antitumor immune response, improving the antitumor activity of CAR T cells.

### 4.4 CAR T cell therapy for ovarian cancer in humans: randomized controlled trials

To date, no randomized controlled trial (RCTs) in humans have been conducted to evaluate the use of CAR T cell therapy for OC. The ClinicalTrials.gov database provides information about 23 ongoing and completed clinical studies in humans receiving CAR T cell therapy for OC. All studies are interventional and use different targets to which CAR T cells are directed, either combined with chemotherapy or not. Of the 23 non-randomized studies, 15 are phase I, three early phase I, and five phase I and II; 12 are designed as single group assignment, seven sequential assignment, and four parallel assignments. RCTs testing CAR T cell therapy in humans with OC are needed in the immediate future, as research in this field is progressing rapidly to improve solid cancer treatment.

There is an ongoing phase 1 clinical trial (NCT05316129) testing maximum tolerated dose and oncological response of intravenous and intraperitoneal autologous CAR T cells targeting the FSHR in patients with recurrent or persistent OC. Despite all clinical trials are not completed or published yet, several reports have been published employing CAR T cell therapy for OC in humans.

Chen et al. explored the safety and feasibility of intravenous anti-MSLN second-generation CAR T cells with a human scFv region in three patients with advanced OC. Two of them reached stabilization of the disease but without achieving partial response, with progression-free survivals of 5.8 and 4.6 months and a CA125 level reduction in serum. None of them experienced cytokine release syndrome or major adverse effects (83).

Fang et al. combined intravenous anti-MSLN CAR T cells secreting anti-PD-1 antibody (αPD-1-mesoCAR-T cells) with apatinib, an anti-angiogenic drug that promotes the infiltration of CD8+ T cells, in a single patient with refractory epithelial OC and liver metastases with recurrent disease after multiline chemotherapy (84). PD-1/PD-L1 inhibition axis has been previously demonstrated to modulate tumor immunosuppressive microenvironment and to enhance antitumor activity of CAR T cells (85). Liver metastatic nodules showed a significant size reduction. The patient achieved partial response and reached a survival of 17 months, with slight adverse effects. CA125 levels in plasma decreased following treatment. These results are probably attributed to the synergic effect of CAR T cell therapy, PD-1/PD-L1 blockade and the antiangiogenic effect of apatinib (84).

Safety and activity of autologous MSLN-targeted second-generation CAR T cells in patients with chemotherapy-refractory pleural mesothelioma, OC and pancreatic carcinoma were explored in a phase I study. One mesothelioma patient developed an anaphylactic reaction likely due to an immune response to the murine scFv region of the CAR. Another patient with metastatic pancreatic adenocarcinoma experienced a dose-limiting toxicity. No on-target toxicity events were observed. Stable disease was the best overall response achieved with the treatment. Only one patient with OC obtained an appreciable but transient reduction in target tumor burden. Prior lymphodepletion with cyclophosphamide resulted in a higher expansion of CAR T cells but not in a longer CAR T cell persistence (86).

According to the rare and heterogeneous available data, CAR T cell therapy seems to be safe in humans but shows poor clinically relevant anti-tumor activity against OC. However, stronger evidence from ongoing and future RCTs is needed.

## 5 Future prospects

### 5.1 CAR-NK cells: an improvement over CAR T cells?

Natural killer (NK) are innate immune cells. As T cells, NKs can also be modified with the introduction of a CAR in order to direct them against tumor cells. CAR NK Cell could have some advantages over CAR T cells. NK do not express MHC so they are unable to induce graft-versus-host disease; therefore, allogeneic or cell lines cells can be infused into patients and not depend solely on autologous cells. Another advantage is that NKs release different cytokine profile which makes them less susceptible to adverse effects such as CRS or neurotoxicity. Also, NKs could show a higher anti-tumor activity because they have multiple mechanisms to target and eliminate cancer cells that are independent of the CAR. Finally, NK have a lifespan of approximately 2 weeks, so in case of adverse effects these would be limited; however, would also require multiple doses to achieve the desired response (87, 88).

Using a murine model, Cao et al. (89) tested the therapeutic effects of MSLN-targeted CAR-engineered natural killer (NK) cells for the treatment of OC. MSLN-targeted CAR-NK cells specifically recognized and killed MSLN-positive OC cells *in vitro*. Intravenous and intraperitoneal infusion induced significant regression of tumor size in both subcutaneous and intraperitoneal tumor models in mice, respectively, and significantly increased the survival of intraperitoneal-tumor-bearing mice.

Another group used third-generation CAR-NK cells targeting CD44 and showed a statistically significant increase over control NK cells in terms of potency and specific cytotoxic activity against CD44-positive OC cell lines and primary OC cells harvested from patient-derived ascites *in vitro*. Interestingly, this group observed that simultaneous treatment with CD44-targeted NK and cisplatin showed higher antitumor activity than sequential treatment (74).

A similar study demonstrated the utility of αFR-targeted CAR-engineered NK cells. The authors employed first-, second-, and third-generation CAR-NK cells in a mouse xenograft model of OC. In *in vitro* studies, the three generations of anti-αFR CAR-NK cells specifically recognized and killed αFR-positive ovarian tumor cells. Third-generation cells showed higher antigen-specific cytotoxicity and proliferation and lower antigen-induced apoptosis compared to first- and second-generation cells, with enhanced antigen-specific degranulation and cytokine secretion. In a peritoneal OC murine model, intraperitoneal injection of third-generation CAR-NK cells notably inhibited the growth of ovarian tumors and significantly prolonged the survival of tumor-bearing mice (73).

Li et al. (90) compared the use of MSLN-targeted CAR-NK cells vs. CAR T cells. CAR-NK cells showed increased antigen-specific cytotoxicity and superior antitumor activity against MSLN-positive OC cells *in vitro*. In a peritoneal OC xenograft model in mice, intraperitoneal CAR-NK therapy was associated with longer persistence of effector cells in peripheral blood, achieved a significant reduction in tumor burden, and markedly improved survival. Moreover, CAR T cell therapy led to a more persistent increase in TNF-α and IL-6 levels, which are tightly related to CRS.

These results suggest that CAR-NK cells could be a better approach than CAR T cells for treatment of OC because of their specific characteristics, as they cause less systemic toxicity due to the absence of IL-6 secretion, which may avoid CRS.

### 5.2 “Stroma first” approach

Solid tumors, including peritoneal metastases, are surrounded by an extracellular stroma containing high levels of collagen and which establishes an important physical barrier against the effects of chemotherapy and penetration of neoplastic cells by CAR T cells (17, 59). However, this collagen matrix can be destroyed using collagenase, facilitating penetration by drugs and CAR T cells and improving the therapy response. The safety and efficacy of peritoneal collagenase in eliminating the tumor stroma by acting as an “enzymatic scalpel” was recently demonstrated by our group in experimental rat and pig models of PC, the findings of which suggest the utility of collagenase lavage of the peritoneal cavity after complete cytoreduction surgery to precondition the peritoneal surface prior to HIPEC administration (59, 60). We further proposed that combined therapy consisting of previous local instillation of collagenase to treat the tumor stroma followed by intraperitoneal CAR T cell administration could improve the efficacy of CAR T cell therapy in PC (13).

## 6 Conclusion

CAR T cells have achieved excellent clinical responses in treating hematological malignancies. In solid tumors, however, the expected results have not materialized. The major obstacle is the accessibility to this therapy. Current costs are very high being unreachable for most patients. Nevertheless, the production of CAR T cells needs no less than 2-3 weeks where tumor progression can occur during this period. Optimizing CAR T cells manufacture and technology must be developed in order to reduce the cost and therefore increase the access to this therapy.

Multiple barriers hinder CAR T cell therapy for OC, including tumor antigen heterogeneity, cell trafficking and durability, antigen loss, the tumor stroma, and the local microenvironment. As in colorectal cancer, intraperitoneally delivered CAR T cells seem more effective than intravenous administration, and this route of administration may facilitate cell migration and durability. Due to antigen heterogeneity, it has been suggested that targeting multiple tumor antigens as part of a dual-target approach (dual CAR T cells and tandem CAR T cells) can avoid antigen escape, improving tumor response and preventing tumor relapse related to antigen loss. TRUCKS have also been proposed to modulate the inhibitory effect of the tumor microenvironment, promoting the antitumor immune response by secreting stimulatory cytokines, which inhibits the suppression of effector T cells. Multiple studies are evaluating new types of CAR T cells and modifying their structure to improve the efficacy, persistence, and trafficking of these cells. NKs seem to be good CAR drivers due to their specific innate characteristics and the absence of IL-6 secretion, which may reduce systemic toxicity by decreasing the risk of CRS; nevertheless, scant research has been published on treating PC, though the results of the few studies that have been conducted are encouraging. A main obstacle facing the treatment of solid tumors is the tumor stroma, indicating that treating the stroma first by means of collagenase administration prior to intraperitoneal CAR T cell infusion can be a promising way to surmount the barriers related to the tumor stroma and the local microenvironment. Thus, peritoneum physiology plays an important role in CAR T cells immunomodulation achieving higher cell migration, durability, and tumor reduction. Improvements in CAR T cells structure are necessary to increase their anti-tumor activity; and lastly, tumor stroma prevent CAR T cells and intraperitoneal chemotherapy to reach tumor cells, hence we believe that the use of collagenase before local treatment will enhance CAR T cell efficacy.

Nevertheless, there is much work to be done in this field before the outcomes of CAR T cell therapy in OC can equal those of hematologic malignancies, and there is little evidence available from randomized controlled trials. The benefit of CAR T cells for the treatment of ovarian malignancies and PC from OC remains unclear. In light of preclinical studies of PC from OC and colorectal cancer, however, this approach holds clear promise.

## Author contributions

VD-P, SQ, CM, SG-S, and PV-C contributed in writing the original draft preparation. VD-P, SQ, CM, and SG-S wrote the manuscript. SQ, PV-C, IG, SJ-G, MGA, HG, and DG-O reviewed the manuscript. SQ and PV-C corrected the manuscript. SQ and PVC edited the manuscript before submission. SQ, PV-C, and DG-O supervised the manuscript. All authors contributed to the article and approved the submitted version.
